# Production of sounds by squirrelfish during symbiotic relationships with cleaner wrasses

**DOI:** 10.1038/s41598-024-61990-8

**Published:** 2024-05-15

**Authors:** Marine Banse, David Lecchini, Justine Sabbe, Noémie Hanssen, Terry Donaldson, Guillaume Iwankow, Anthony Lagant, Eric Parmentier

**Affiliations:** 1 Laboratoire de Morphologie Fonctionnelle Et Evolutive, FOCUS, Université de Liège, 4000 Liège, Belgium; 2 EPHE-UPVD-CNRS, USR 3278 CRIOBE, PSL University, Moorea, French Polynesia; 3 Laboratoire d’Excellence “CORAIL”, 58 Avenue Paul Alduy, 66860 Perpignan, France; 4 University of Guam Marine Laboratory/Guam EPSCoR, UOG Station, Mangilao, Guam 96923 USA

**Keywords:** Acoustic communication, Cleaner fish, Agonistic interactions, Holocentridae, Zoology, Animal behaviour

## Abstract

Examples of symbiotic relationships often include cleaning mutualisms, typically involving interactions between cleaner fish and other fish, called the clients. While these cleaners can cooperate by removing ectoparasites from their clients, they can also deceive by feeding on client mucus, a behavior usually referred to as “cheating behavior” that often leads to a discernible jolt from the client fish. Despite extensive studies of these interactions, most research has focused on the visual aspects of the communication. In this study, we aimed to explore the role of acoustic communication in the mutualistic relationship between cleaner fishes and nine holocentrid client species across four regions of the Indo-Pacific Ocean: French Polynesia, Guam, Seychelles, and the Philippines. Video cameras coupled with hydrophones were positioned at various locations on reefs housing Holocentridae fish to observe their acoustic behaviors during interactions. Our results indicate that all nine species of holocentrids can use acoustic signals to communicate to cleaner fish their refusal of the symbiotic interaction or their desire to terminate the cooperation. These sounds were predominantly observed during agonistic behavior and seem to support visual cues from the client. This study provides a novel example of acoustic communication during a symbiotic relationship in teleosts. Interestingly, these vocalizations often lacked a distinct pattern or structure. This contrasts with numerous other interspecific communication systems where clear and distinguishable signals are essential. This absence of a clear acoustic pattern may be because they are used in interspecific interactions to support visual behavior with no selective pressure for developing specific calls required in conspecific recognition. The different sound types produced could also be correlated with the severity of the client response. There is a need for further research into the effects of acoustic behaviors on the quality and dynamics of these mutualistic interactions.

## Introduction

Communication is an intricate phenomenon involving the exchange of information between two or more individuals, who use information to take a decision^1^. For communication to be deemed effective, it should influence the behavior of the receiver, leading to advantageous outcomes at least for the sender, and eventually to the recipient. Teleost fishes undoubtedly constitute the largest group of sound-producing vertebrates, having evolved a diverse array of mechanisms^2^ for generating vocalizations that play a pivotal role in facilitating social interactions. The fact that this ability has evolved at least 33 times^3^ supports the importance of the acoustic channel in fish behavior^4^.

Most of the reported behaviors related to acoustic communication concern sensu lato agonism^5,6^ and reproduction^7,8^. During reproductive behaviors, sounds play essential roles in recognizing and selecting potential sexual partners, indicating readiness to spawn, assessing male fitness, and synchronizing gamete release^9–11^. During intra- or interspecific agonistic relationships, sounds can be used to defend a territory including resources and to protect eggs^12^ or nests^13,14^. Some of the agonistic calls can be considered as alarm or distress calls when they are produced by fish at the approach of a predator or while they are manipulated^15^. They can also be used to warn or deter predators in case of aposematism^16,17^. It is worth mentioning that the codification of the signal could be less accurate in heterospecific communication since it should be interpreted by different species^14,18^. The ability to generate diverse kinds of sounds is intrinsic to each species. Some emit identical sounds in various behavioral contexts^12^, other species produce distinct sounds based on the context^10,14,19^.

In Holocentridae (soldierfish and squirrelfish), studies focusing on sounds have been conducted on a few species^20–26^. Sounds can be produced in different behavioral contexts, including territorial defence, agonistic interactions, and alarm calls. Unfortunately, the authors seem to have used different terms to describe the same sounds or reported different types of sounds for similar behaviors, challenging the establishment of clear relationships between the type of sound and the behavior.

A prominent mutualistic association on coral reefs is between cleaner fishes and their clients who approach them for ectoparasite removal^27^. This relationship is crucial as it provides a food source for the cleaners and enhances the fitness and survival of the clients when relieved of their parasites^28^. However, the cleaners have a preference for feeding on the mucus or tissues of the clients, highlighting the vague boundaries between the different types of the symbiotic interactions^29^. The temptation to cheat is therefore significant and constitutes an integral part of this cooperation^30^, where clients have to coax the cleaners into feeding against their preference^31^. The act of cheating causes the clients to prematurely end the interaction by chasing the cleaner, effectively punishing it through the denial of a food resource^27,32^. Nevertheless, the aggressive pursuit by the client brings future benefits as cheating becomes less frequent in subsequent interactions when the cleaner has previously been punished through aggressive behavior^33^. These observations suggest that the cleaners learn to recognize their clients^34^. By punishing undesirable behavior while promoting positive interactions, the control of the interaction ensures a better quality of cleaning service by the cleaner^30,31^. Since different holocentrids can make sounds, our study aims to investigate the potential role of acoustic communication in the relationship between cleaners and their clients. The aims of this study were to (1) highlight acoustic communication in interspecific relationships between cleaner fishes and clients and (2) describe the acoustical variables of the sounds produced by the clients in relation to the interaction.

## Results

The 190 video recordings of the interactions between the nine Holocentridae species (*Myripristis violacea, M. berndti, M. kuntee, Neoniphon sammara, N. diadema, N. microstoma, Sargocentron seychellense, S. spiniferum, S. caudimaculatum*) and their three cleaner species (*Labroides bicolor*, *L. dimidiatus* and *L. rubrolabiatus*) revealed distinct patterns of heterospecific behavior. Different situations were observed (Suppl. Videos S1–S4). In some cases, the client fish rejected the interaction and simultaneously produced sounds. When interactions were accepted, these encounters often lasted only a few seconds before the holocentrids abruptly ended the cleaning process. In some instances, this termination was followed by the holocentrids pursuing the individual *Labroides* over varying distances (Suppl. Videos S3 and S4). Alternatively, the holocentrids left the scene. All negative interactions (the initial rejection, the termination and the subsequent chase) can be accompanied by sounds. Notably, among all these interactions, only the holocentrids were observed to produce sounds.

**Figure 1:**
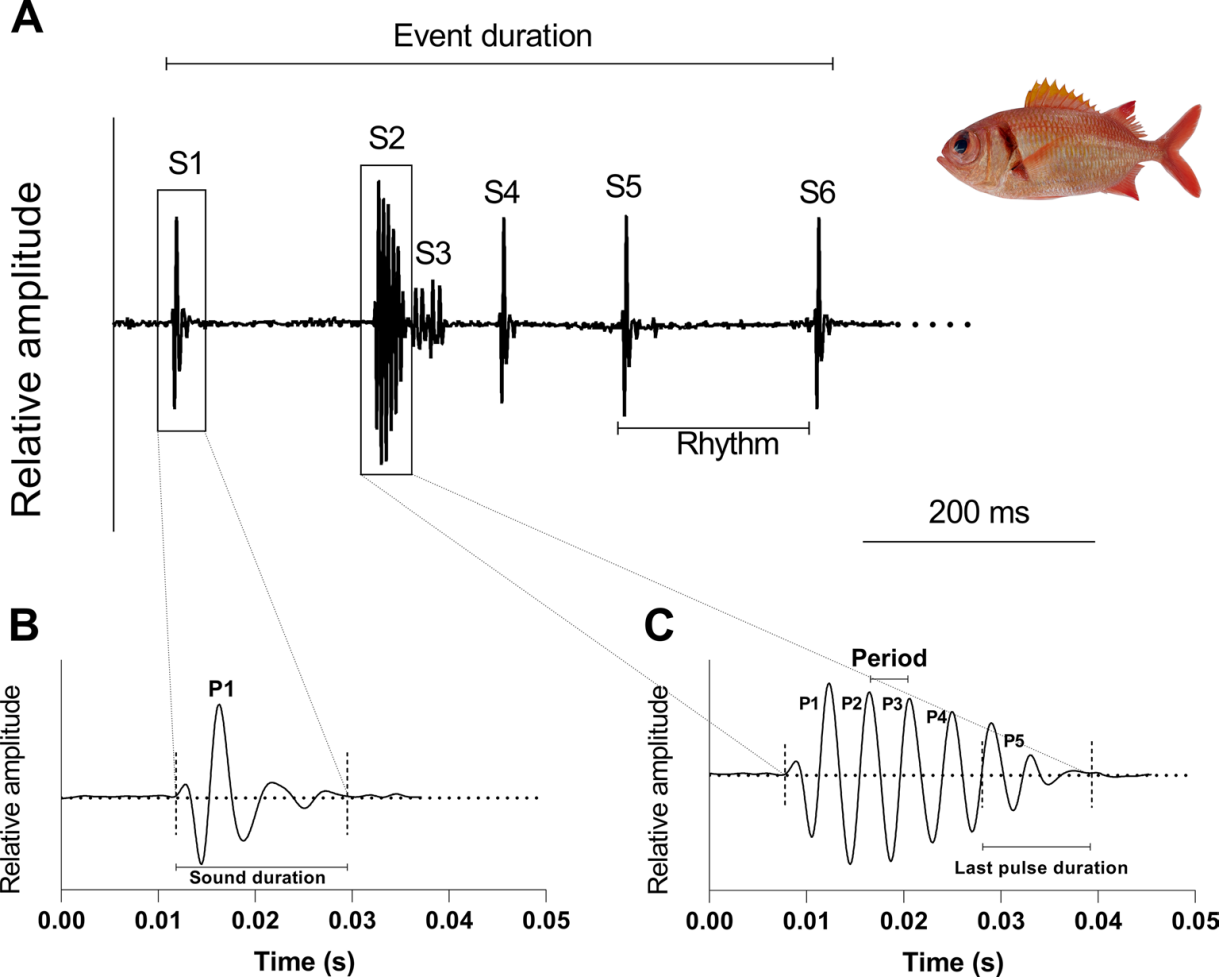
Oscillograms showing an acoustic event made by *Myriprisits kuntee* during an agonistic interaction with a cleaner fish *Labroides* (**A**). The different panels provide information on the way the different temporal features were measured: duration of the event, number of sounds (S) composing the event, rhythm, sound duration, number of pulses (P) in the sound, pulse periods, duration of the last pulse. In this example, the acoustic event is made of 6 sounds (S1-S6). Note that sounds can be composed of a single pulse (as observed in S1, S4, S5, and S6) or multiple pulses (as seen in S2 and S3), with the latter essentially being repetitions of a singular pulse. The magnification of S1 in (B) illustrates a sound consisting of a single pulse, while the magnification of S2 in (C) displays a sound composed of several pulses.

**Figure 2:**
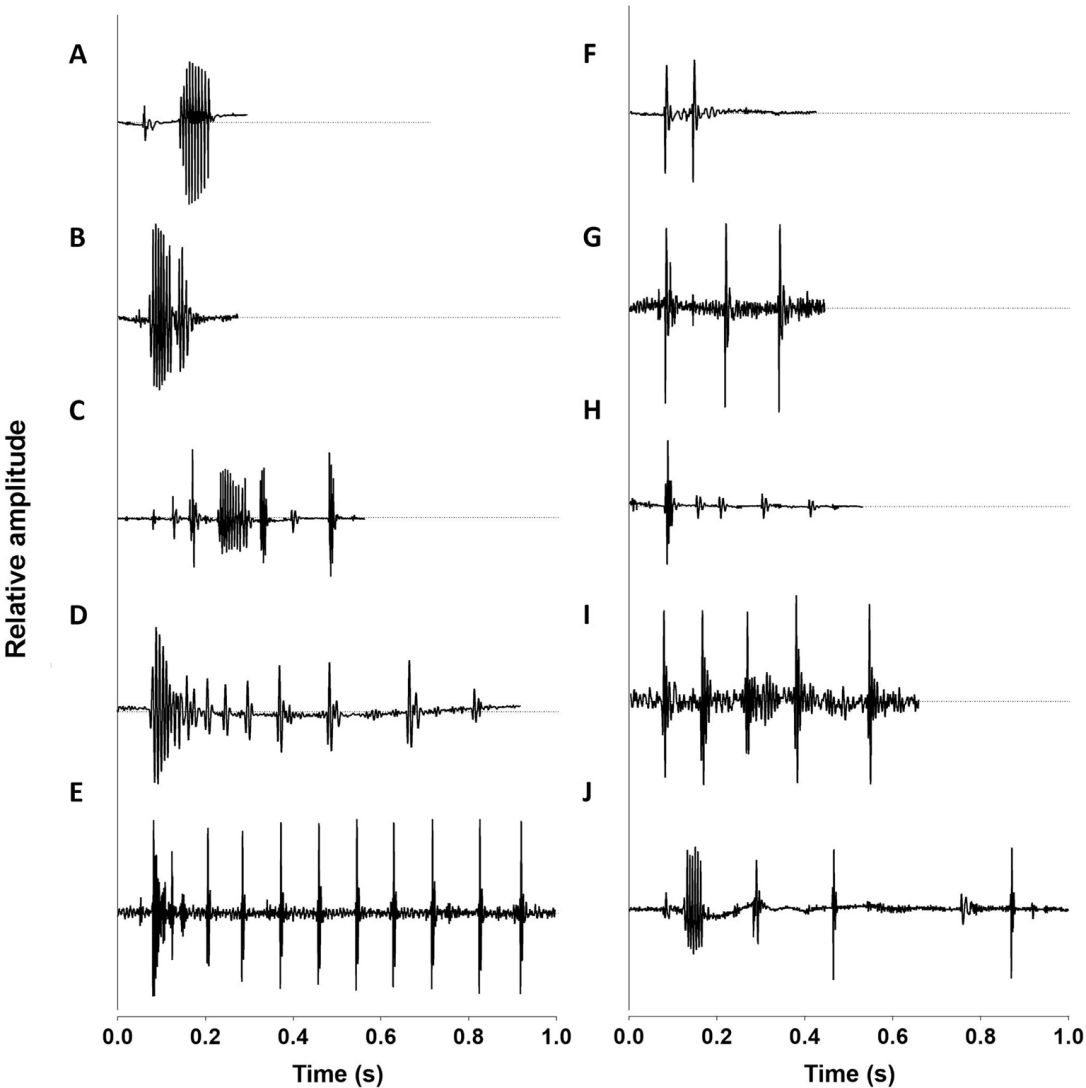
Example of different acoustic event*s* produced by *Myriprisits kuntee* during agonistic interaction*s* with cleaner fishes *L. dimidiatus, L. bicolor and L. rubrolabiatus*. These examples illustrate the absence of a fixed vocal pattern associated with the interaction and clearly shows that an event can be made of different kinds of sounds.

For the purpose of this study, we focused our analyses on species whose the total number of observed acoustic behaviors was at least fifteen and for which the sounds showed a good signal-to-noise ratio. The analysis of the videos revealed 52 acoustic behaviors in *M. violacea*, 15 in *M. kuntee*, 20 in *N. sammara*, and 27 in *S. seychellense*. For each of the four species, we analyzed the production of acoustic events that refer to a sonic behavioral interaction between a holocentrid and a cleaner fish specimen (Fig. 1). The considerable variability observed in acoustic parameters for all holocentrid species and their cleaning partners significantly challenged the applicability and relevance of statistical tests. Each event was composed of one to several sounds, themselves composed of one to several pulses (Figs. 1 and 2). For all species, acoustic events produced during agonistic interactions greatly vary in terms of duration, number of sounds and rhythm (Table 1) which is here defined as the time interval between the onset of two consecutive sounds (Fig. 1).

**Table 1:** Summary of the acoustical variables (mean ± sd and [min–max values]) related to acoustic events produced by four holocentrid species during agonistic interactions with cleaner fishes *L. dimidiatus*, *L. bicolor* and *L. rubrolabiatus*.

Species	Event duration (ms)	Number of sounds/event	Rhythm (ms)
*M. kuntee* (N = 15)	393 ± 418 [8.9–1653]	2.9 ± 1.6 [1–6]	193 ± 220 [12–825]
*M. violacea* (N = 52)	310 ± 523 [4–2621]	3 ± 3.2 [1–18]	141 ± 118 [14–739]
*N. sammara* (N = 20)	288 ± 248 [19–792]	2.5 ± 1.2 [1–6]	182 ± 127 [20–509]
*S. seychellense* (N = 27)	234 ± 317 [10–1270]	2 ± 1.34 [1–5]	217 ± 147 [51–636]

(N) refers to the number of events analyzed for each species.

Therefore, it seems that this particular heterospecific acoustic communication lacks a stereotyped pattern and appears rather random (Fig. 2). Sounds composing these acoustic events were not stereotyped either. All species produced sounds that lasted on average 14–20 ms and were mainly made of 1 pulse (Table 2). However, some of these sounds were made of 2–19 pulses. Overall means are similar among species and show overlap between all four species for sound duration, number of pulses per sound, pulse period and duration of the last pulse. The variation in parameters was greater in *Myripristis* than in *Neoniphon* and *Sargocentron* species. For instance, pulse period varies from 7 to 9 ms and 8 to 11 ms in *N. sammara* and *S. seychellense*, respectively, but from 4 to 20 ms in *M. kuntee* and *M. violacea*. A similar observation can be made for the duration of the last pulse (Table 2). Finally, fundamental and dominant frequencies ranged, respectively, between 128 and 260 Hz, and 76 and 869 Hz, for all species.

**Table 2:** Summary of the acoustical variables (mean ± SD and [min–max values]) of sounds produced by four holocentrid species during agonistic interactions with cleaner fishes.

Species	Sound duration (ms)	Number of pulses	Pulse period (ms)	Fundamental frequency (Hz)	Dominant frequency (Hz)	Last pulse duration (ms)
*M. kuntee* (n = 44)	14.3 ± 21.4 [3.4–147.5]	1.5 ± 1.3 [1–8]	10.6 ± 6.5 [3.9–26.5]	250 ± 14 [240–260]	273 ± 103 [152–621]	9.8 ± 5.3 [4.4–20.3]
*M. violacea* (n = 154)	21.5 ± 23.5 [4–138.5]	2.3 ± 3.4 [1–19]	7.3 ± 3.3 [3.4–21]	172 ± 17 [128–192]	230 ± 116 [76–841]	10.8 ± 4.7 [4.4–22.3]
*N. sammara* (n = 50)	14.2 ± 5 [6–34.1]	1.1 ± 0.4 [1–4]	7.5 ± 1.1 [6.7–8.7]	140 [140–140]	325 ± 181 [146–869]	11.7 [11.7–11.7]
*S. seychellense* (n = 54)	13.9 ± 5 [4.5–33.5]	1.1 ± 0.4 [1–3]	9.7 ± 1.3 [8–11.4]	140 [140–140]	283 ± 35 [216–386]	12.4 ± 2.3 [10.6–15.7]

(n) refers to the number of sounds analyzed for each species.

## Discussion

Instances of effective cooperation and cheating have been documented in interactions between cleaners, such as *Labroides* species, and their client fish. When cleaners opt for dishonesty, it leads to conflict, manifested by sudden, and abrupt movements from the client^35–37^. In our study, holocentrid fishes sometimes pursued *Labroides* specimens, punishing the cleaner through the denial of a food resource^30,35^. This shows that communication is an intricate phenomenon involving the exchange of information between two or more individuals who use that information to take a decision^1^. During vocal intraspecific communication, the message is encoded in acoustic features, influencing the behavior of the receiver and leading to advantageous outcomes, at least for the sender, and potentially for the recipient. The codification of the signal could be less accurate in heterospecific communication since it should be interpreted by different species^14,18^.

In the cleaner-client interaction, stereotyped visual communication is known to precede the cooperation. Cleaners actively assert their intentions to clean, often by using conspicuous dances, or through tactile stimulation^27,38^. Interested clients can then pose to signal a desire to be cleaned. Visual communication also appears to be important when ending a cleaning interaction, but it seems to be less stereotyped since clients simply twitch to indicate their desire to break the interaction, or they may also simply depart by swimming away^39^. Our video analyses revealed that interactions between holocentrid species and cleaners are not solely visual; they can also be accompanied with the production of sounds by the holocentrid clients (Suppl. Videos S1–S4). This observation is further confirmed by the sound features, which align with holocentrid sounds recorded in previous studies^21,24–26^. The cleaner fish, on the other hand, did not produce any sounds, consistent with observations that members of that family (Labridae) do not encompass many vocal species^25,40^. These findings were consistent across a vast geographic area (French Polynesia, Seychelles, Guam, and Philippines) and were based on observations from 64 videos (and 190 interactions) featuring various client fish and cleaners. In the framework of the client–cleaner interaction, nine different holocentrid species produced sounds towards three different cleaner species.

The sounds produced by holocentrids during interactions with cleaners represent a novel kind of acoustic interspecific communication, in which the client has the control, determining the course of the interaction. While pomacentrids are capable of producing varied sounds depending on the behavioral context, no sounds from the damselfish *Dascyllus aruanus* have been documented during cleaning interactions where this pomacentrid acted as the cleaner^41^. As the sounds in holocentrids were always associated with host body movements, they are most probably used to reinforce a visual behavior, namely a lateral twitch executed by the fish. The get-out signal we recorded, used either before or to terminate an interaction, did not seem to be stereotypical, as it consisted of either a single type or a combination of sounds produced at irregular intervals. The lack of specificity in the sound and in sonic pattern within a species could be explained in different ways. Sounds are used in interspecific interactions and support visual behavior, meaning there is no pressure for developing specific calls. In the same way, the stridulatory sounds of different catfish species facing predators^42–44^ can be highly diverse. This is most probably because they do not have a role in behaviors requiring conspecific recognition, such as reproduction^17^. Recently, it has been reported that holocentrids can produce different kinds of sounds suggesting their ability to provide varied information based on the perceived predation risk. They exhibit an acoustic mobbing behavior when a predator approaches but emit a different sound when captured^45^. In this study, we focused on the qualitative aspect, demonstrating that acoustic communication can occur in at least nine species of holocentrids during client-cleaning interactions. This finding supports the hypothesis that such behavior is prevalent across all species within the holocentrid family.

However, there is information we lack that deserves future studies. According to Bshary and Grutter^32^, clients must make cleaners feed on ectoparasites as expected in this type of interaction, and not on protective mucus, for the service to be beneficial to the client. It means that the ability of a client to control the course of an interaction, either by avoiding or by terminating an exchange, should affect service quality^31^. Moreover, eavesdropping clients spend more time alongside 'cooperative' cleaners compared to cleaners with an ‘unknown level of cooperation’, which shows that clients engage in image-scoring behavior^32^. Since the interaction can conclude either silently or with various types of sounds, it is essential to investigate whether the client metes out varying degrees of punishment based on these sounds and to examine its subsequent impact on cleaner behavior. In this way, studies in controlled environment should investigate whether the acoustic pattern can be related to the client’s motivation in response to the cleaner’s behavior and whether sound affects the behavior of cleaners to improve their image quality in the eyes of eavesdropping clients. Experiments should also be conducted using other acoustic species, such as pomacentrids, triggerfish or chaetodontids, to know whether they can also use sounds in this kind of symbiotic relationships.

## Materials and methods

### Study area and subjects

Video recordings took place during daylight in 4 regions of the Indo-Pacific Ocean (French Polynesia, Guam, Seychelles and Philippines) between October 2020 and July 2022: (1) Opunohu Bay, Moorea Island, French Polynesia: October–November 2020; (2) Tumon Bay, Guam, USA : December 2021; (3) Mahé Island, Seychelles: February March 2022; (4) Dauin (9° 11′ N, 123° 16′ E), Negros Island, Philippines : July 2022.

The recordings were conducted on 64 sites for a total of approximately 77 h of videos (Table S1). A session corresponds to a video recording made in front of a shelter used by Holocentridae. The duration of a session varied from 27 to 179 min. The recordings were all made between sunrise and sunset. As no specimens were taken from the environment, this study did not require a permit.

From the recordings, 9 species of Holocentridae from 3 genera (*M. violacea, M. berndti, M. kuntee, N. sammara, N. diadema, N. microstoma, S. seychellense, S. spiniferum, S. caudimaculatum*) performed 190 behaviors corresponding to an acoustic interaction with cleaner fishes from the family Labridae (*L. bicolor*, *L. dimidiatus* and *L. rubrolabiatus*). All recorded specimens were adults. Sex are not classifiable in these species. For the purpose of this paper, our analyses focused on interactions for which the total number of observed acoustic behaviors was at least fifteen and for which the sounds showed a good signal-to-noise ratio (N = 114). Acoustic interactions involved *M. violacea* (N = 52), *M. kuntee* (N = 15), *N. sammara* (N = 20), *S. seychellense* (N = 27).

### Field procedures

Recordings were made as 44.1 kHz 16-bit WAV files on digital audio recorders. Recording devices (Spy-fish, Liège, Belgium) consisted of a modified GoPro 6 (GoPro, San Mateo, CA, USA) inserted into a waterproof case and coupled to an external hydrophone HTI 96-Min (High Tech Inc., Long Beach, MS, USA, frequency range: 20 Hz–20 kHz, sensitivity: − 164 dBV mPa^−1^). The systems were either positioned directly on the seabed or placed on a tripod, at approximately 1 m away from the caves used by the Holocentrids. We placed the cameras and then left the area under recording to avoid any external disturbance likely to modify the behaviors of the fishes.

### Video analysis

A total of about 77 h of videos were recorded. Within the selected coral reefs for this study, several species of Holocentridae coexist, belonging to the genera *Myripristis*, *Neoniphon*, and *Sargocentron*. The study was conducted in two phases. In the initial phase, two researchers independently observed the video recordings to catalog behavioral events associated with sound production across all encountered Holocentridae species. This approach by several independent observers has the great advantage of increasing the reliability of the observations. All the videos were analyzed using DaVinci Resolve (version 1.3.2). During the viewing process, various colored markers were used to differentiate the Holocentridae species on the video tapes, allowing for the identification and positioning of all behaviors associated with sounds on the tapes. These were then viewed a second time to verify and classify the different behaviors. Only the behaviors involving a holocentrid (client fish) and its cleaner were kept for further analysis. We kept the videos where the emitting species could be identified. In the case of the client–cleaner relationship, we kept the videos where the production of sounds was accompanied by a movement of the fish's body. The observations revealed that the sounds were always emitted by Holocentridae, not by the cleaner. The results were then cross-referenced, and ambiguous cases (species identification, sound-behavior association, sound emitter identification, etc.) were either retained or discarded from the study to avoid any bias. Hardly recognizable species were not taken into account in this study (e.g. *Myripristis pralinia*, *M. murdjan*, *M. amaena*).

### Sound analysis

Soundtracks were extracted from the videos on DaVinci Resolve and sounds were isolated from these soundtracks before being manually investigated using the software Avisoft-SAS Lab Pro 5.2.13 (Avisoft Bioacoustics, Glienicke, Germany). All recordings were digitized at 48 kHz (16-bit resolution). Before analysis, low pass filtered (1000 Hz) was applied. Analysis focused first on the acoustic events and then on the sounds. An acoustic event refers to a sonic behavioral interaction between an holocentrid specimen and a cleaner fish specimen. Each event can be composed of one to several distinct sounds and each of these sounds can be composed of one to several pulses. Temporal features were measured from oscillograms whereas frequencies were obtained from logarithmic power spectra. Several acoustical parameters were measured: (1) duration of the event (ms), (2) number of sounds composing the event, (3) rhythm (also called “inter onset interval”, defined as the time interval between the onset of two consecutive sounds, ms), (4) sound duration (ms), (5) number of pulses in the sound, (6) pulse periods (measured as the peak-to-peak intervals between two consecutives pulses, ms), (7) duration of the last pulse (ms) based on oscillograms (Fig. 1), (8) fundamental frequency (Hz) and (9) dominant frequency (defined as the frequency with the highest energy, Hz) of the complete sound. Depending on the number of pulses in the sound, some variables could not be measured for some sounds (e.g.: no fundamental frequency for sounds made of 1 or 2 pulses).

### Supplementary Information

Supplementary Video S1.

Supplementary Video S2.

Supplementary Video S3.

Supplementary Video S4.

Supplementary Legends.

Supplementary Table S1.

## Data Availability

The data are available from the corresponding author upon reasonable request.
