# Author Correction: Production of sounds by squirrelfish during symbiotic relationships with cleaner wrasses

**DOI:** 10.1038/s41598-024-67937-3

**Published:** 2024-07-30

**Authors:** Marine Banse, David Lecchini, Justine Sabbe, Noémie Hanssen, Terry Donaldson, Guillaume Iwankow, Anthony Lagant, Eric Parmentier

**Affiliations:** 1 Laboratoire de Morphologie Fonctionnelle Et Evolutive, FOCUS, Université de Liège, 4000 Liège, Belgium; 2 EPHE-UPVD-CNRS, USR 3278 CRIOBE, PSL University, Moorea, French Polynesia; 3 Laboratoire d’Excellence “CORAIL”, 58 Avenue Paul Alduy, 66860 Perpignan, France; 4 University of Guam Marine Laboratory/Guam EPSCoR, UOG Station, Mangilao, Guam 96923 USA

Correction to: *Scientific Reports* 10.1038/s41598-024-61990-8, published online 15 May 2024

The original version of this Article contained errors in the names of the authors Marine Banse, David Lecchini, Justine Sabbe, Noémie Hanssen, Terry Donaldson, Guillaume Iwankow, Anthony Lagant & Eric Parmentier, which were incorrectly given as Banse Marine, Lecchini David, Sabbe Justine, Hanssen Noémie, Donaldson Terry, Iwankow Guillaume, Lagant Anthony & Parmentier Eric.

The Author Contribution section now reads:

“M.B. and E.P. conceptualized the study, supervised the project and wrote the original draft. Investigation and visualization were performed by M.B., with assistance in collect by D.L., T.D., A.L. and G.I. Sound and video analysis were performed by M.B., N.H. and J.S. E.P., D.L. and T.D. acquired funding. All authors reviewed the manuscript.”

The original Article has been corrected.

