# Supplementary material for: Production of sounds by squirrelfish during symbiotic relationships with cleaner wrasses
Source: Sci Rep. 2024 May 15;14:11158. doi: 10.1038/s41598-024-61990-8 (PMC11096179; doi:10.1038/s41598-024-61990-8)
Supplement: Supplementary file 5 — Supplementary Legends. [file 41598_2024_61990_MOESM5_ESM.docx]

**Supplementary video legends**

**Video S1.** This sequence captures *Myripristis violacea* declining the interaction and actively chasing away the cleaner fish, *Labroides bicolor*. This footage was taken in French Polynesia.

**Video S2.** This sequence captures *Sargocentron seychellense* declining the interaction and actively chasing away the cleaner fish, *Labroides dimidiatus*. This footage was taken in Seychelles.

**Video S3.** This sequence captures *Neoniphon sammara* declining the interaction with the cleaner fish, *Labroides bicolor*. This footage was taken in French Polynesia.

**Video S4.** This sequence captures *Neoniphon sammara* terminating the interaction with the cleaner fish, *Labroides bicolor*. This footage was taken in French Polynesia.
