# Supplementary material for: Production of sounds by squirrelfish during symbiotic relationships with cleaner wrasses
Source: Sci Rep. 2024 May 15;14:11158. doi: 10.1038/s41598-024-61990-8 (PMC11096179; doi:10.1038/s41598-024-61990-8)
Supplement: Supplementary file 6 — Supplementary Table S1. [file 41598_2024_61990_MOESM6_ESM.docx]

**Table S1**. Recording sessions conducted in the 4 regions. This table lists the assigned numbers for various recording sessions, the study region, the precise location within the regions, the numbers assigned to the fish shelters where the cameras were positioned, the total duration of each session (in minutes), and the date they were recorded.

| **Session** | **Region** | **Location** | **# shelter** | **Recording time (min)** | **Date** |
| --- | --- | --- | --- | --- | --- |
| 1 | **Guam** | Agat | 1 | 163 | 13/12/2021 |
| 2 |  | Fish Eye | 1 | 61 | 27/11/2021 |
| 3 |  | Tumon Bay | 1 | 75 | 01/12/2021 |
| 4 |  | Tumon Bay | 2 | 79 | 01/12/2021 |
| 5 |  | Tumon Bay | 3 | 79 | 04/12/2021 |
| 6 |  | Tumon Bay | 4 | 103 | 05/12/2021 |
| 7 |  | Tumon Bay | 5 | 65 | 05/12/2021 |
| 8 |  | Tumon Bay | 6 | 55 | 08/12/2021 |
| 9 |  | Tumon Bay | 7 | 96 | 08/12/2021 |
| 10 |  | Tumon Bay | 8 | 55 | 08/12/2021 |
| 11 |  | Tumon Bay | 9 | 48 | 08/12/2021 |
| 12 |  | Tumon Bay | 10 | 27 | 11/12/2021 |
| 13 |  | Tumon Bay | 13 | 50 | 11/12/2021 |
| 14 | **French Polynesia** | Papetoai | 1 | 89 | 19/11/2020 |
| 15 |  | Papetoai | 2 | 93 | 06/11/2020 |
| 16 |  | Papetoai | 3 | 76 | 06/11/2020 |
| 17 |  | Papetoai | 4 | 67 | 19/11/2020 |
| 18 |  | Tiahura | 1 | 70 | 25/11/2020 |
| 19 |  | Papetoai | 6 | 64 | 17/11/2020 |
| 20 |  | Piha’ena | 1 | 101 | 04/11/2020 |
| 21 |  | Temae | 1 | 74 | 24/11/2020 |
| 22 |  | Haapiti | 1 | 61 | 01/12/2020 |
| 23 |  | Haapiti | 2 | 97 | 16/11/2020 |
| 24 |  | Haapiti | 3 | 70 | 23/11/2020 |
| 25 |  | Tiahura | 2 | 88 | 24/11/2020 |
| 26 |  | Tiahura | 3 | 88 | 27/11/2020 |
| 27 |  | Piha’ena | 1 | 75 | 04/11/2020 |
| 28 |  | Cook’s Bay | 2 | 58 | 14/10/2020 |
| 29 |  | Haapiti | 4 | 80 | 01/12/2020 |
| 30 |  | Haapiti | 5 | 79 | 29/10/2020 |
| 31 |  | Papetoai | 5 | 71 | 06/11/2020 |
| 32 |  | Papetoai | 6 | 81 | 06/11/2020 |
| 33 |  | Papetoai | 7 | 62 | 17/11/2020 |
| 34 |  | Papetoai | 8 | 62 | 17/11/2020 |
| 35 |  | Papetoai | 9 | 44 | 19/11/2020 |
| 36 |  | Papetoai | 10 | 30 | 19/11/2020 |
| 37 |  | Temae | 2 | 64 | 24/11/2020 |
| 38 |  | Tiahura | 1 | 40 | 30/10/2020 |
| 39 |  | Tiahura | 4 | 70 | 24/11/2020 |
| 40 |  | Tiahura | 5 | 76 | 25/11/2020 |
| 41 |  | Tiahura | 6 | 90 | 27/11/2020 |
| 42 |  | Piha’ena | 2 | 34 | 09/10/2020 |
| 43 |  | Piha’ena | 3 | 79 | 30/10/2020 |
| 44 | **Seychelles** | FairyLand | 1 | 77 | 05/04/2022 |
| 45 |  | FairyLand | 2 | 65 | 05/04/2022 |
| 46 |  | FairyLand | 3 | 61 | 05/04/2022 |
| 47 |  | FairyLand | 4 | 59 | 06/04/2022 |
| 48 |  | FairyLand | 5 | 62 | 06/04/2022 |
| 49 |  | FairyLand | 6 | 66 | 06/04/2022 |
| 50 |  | FairyLand | 7 | 66 | 06/04/2022 |
| 51 |  | FairyLand | 8 | 63 | 08/04/2022 |
| 52 |  | FairyLand | 9 | 67 | 08/04/2022 |
| 53 |  | FairyLand | 10 | 60 | 08/04/2022 |
| 54 |  | FairyLand | 11 | 29 | 08/04/2022 |
| 55 |  | FairyLand | 12 | 61 | 08/04/2022 |
| 56 |  | Sunset | 1 | 61 | 12/04/2022 |
| 57 |  | Sunset | 2 | 66 | 12/04/2022 |
| 58 |  | Sunset | 3 | 57 | 12/04/2022 |
| 59 |  | FairyLand | 13 | 71 | 13/04/2022 |
| 60 |  | FairyLand | 14 | 70 | 13/04/2022 |
| 61 |  | Sunset | 1 | 64 | 27/03/2022 |
| 62 |  | Sunset | 2 | 62 | 27/03/2022 |
| 63 | **Philippines** | Dauin | 1 | 173 | 07/07/2022 |
| 64 |  | Dauin | 2 | 179 | 07/07/2022 |
